# Antibacterial polyphenols for osteomyelitis treatment: from antibacterial mechanisms to biomaterial-based delivery

**DOI:** 10.3389/fcell.2026.1943374

**Published:** 2026-09-03

**Authors:** Xuemei Cao, Yuanmin He, Yamin Zhu, Ping Gu, Ting Dai, Yuyu Zheng

**Affiliations:** 1 Department of Dermatology, Qionglai City Medical Center Hospital, Qionglai, Sichuan, China; 2 Department of Dermatology, The Affiliated Hospital of Southwest Medical University, Luzhou, Sichuan, China

**Keywords:** antibacterial polyphenols, biofilm, biomaterials, bone regeneration, microenvironmental adaptation, osteomyelitis

## Abstract

Osteomyelitis is a refractory infection caused by bacterial invasion of bone tissue. Biofilms, small-colony variants (SCVs), and intracellular persistence enable bacteria to evade immune clearance and antibiotic killing, while sequestra and implant surfaces may act as long-term infection reservoirs. Some pathogens can also invade the osteocyte lacuno-canalicular network, further hindering complete eradication. Meanwhile, persistent inflammation, oxidative stress, and impaired local blood supply aggravate bone destruction and inhibit tissue repair. Therefore, osteomyelitis treatment requires not only infection control, but also the reduction of inflammatory damage, promotion of angiogenesis, and enhancement of bone regeneration. Natural polyphenols exhibit multitarget antibacterial and antibiofilm activities by disrupting bacterial membranes and cell walls, interfering with key enzymes and energy metabolism, regulating metal homeostasis, and increasing bacterial susceptibility to antibiotics. They also possess anti-inflammatory and redox-regulatory properties and can modulate osteogenesis and bone resorption. Their abundant phenolic hydroxyl groups and coordination sites facilitate their integration with metal ions, polymers, and inorganic materials to construct local delivery systems. Nanoparticles, coatings, hydrogels, and bone scaffolds can improve the retention and release of polyphenols at infected sites and enable infection-responsive or stage-specific treatment. These biomaterial-based strategies may adapt to the acidic, hypoxic, and persistently inflamed osteomyelitis microenvironment, coordinating antibacterial activity, immune regulation, and bone regeneration. This mini-review summarizes the major classes and structural features of natural polyphenols, their antibacterial mechanisms, and biomaterial-based delivery strategies, with emphasis on microenvironmental adaptation and clinical translation in osteomyelitis treatment.

## Highlights

- Natural polyphenols exert multitarget antibacterial and antibiofilm effects.
- Biomaterial integration improves local retention and controlled release.
- Responsive systems adapt to the osteomyelitis microenvironment.

## 1 Introduction

**FIGURE 1:**
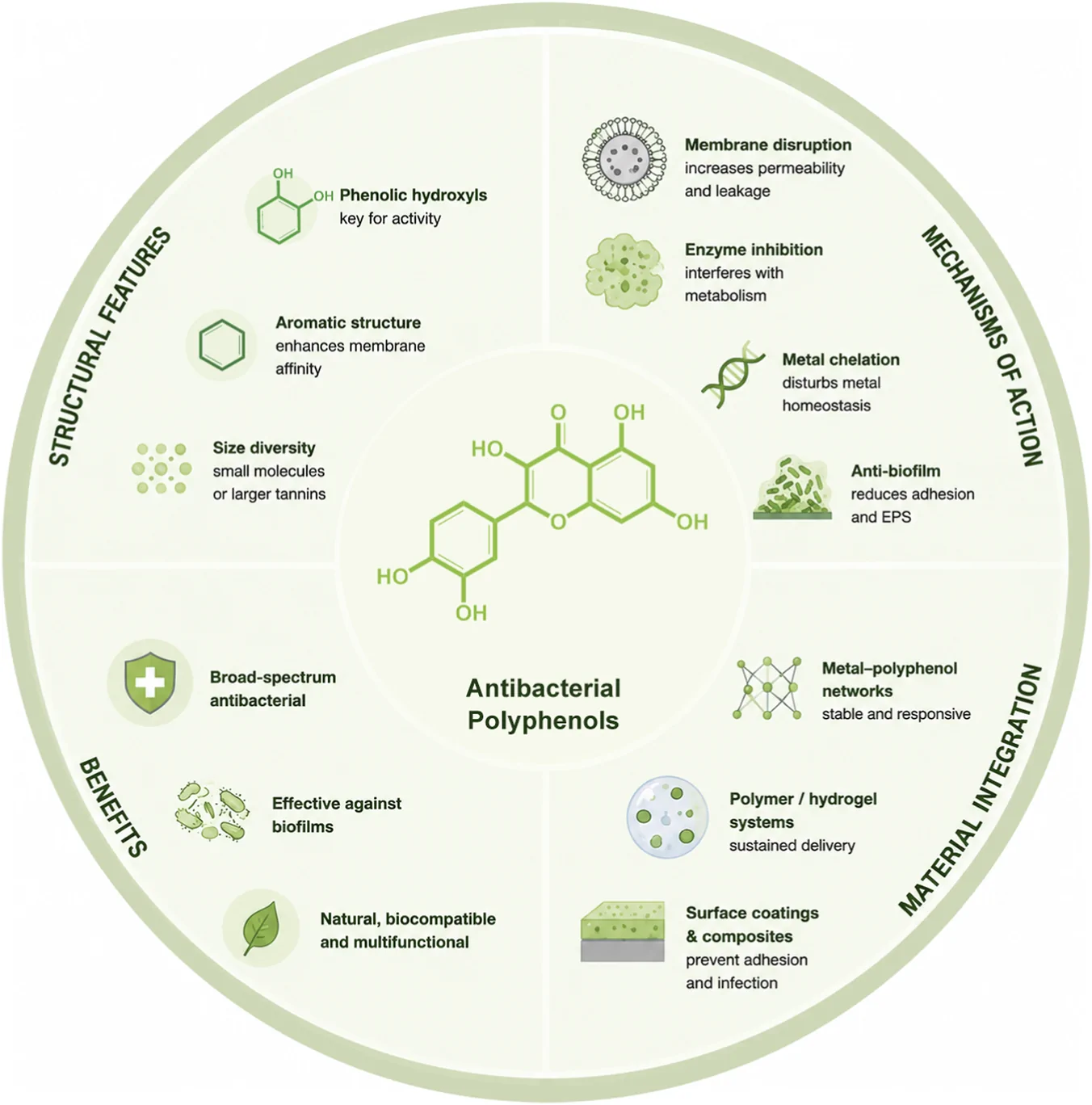
Structural characteristics, antibacterial mechanisms, therapeutic benefits, and biomaterial integration strategies of antibacterial polyphenols.

Osteomyelitis can result from hematogenous spread, open injuries, orthopedic surgery, or infection of adjacent soft tissues (Kremers et al., 2015). *Staphylococcus aureus* is the most common causative pathogen, and the emergence of methicillin-resistant *S. aureus* and other drug-resistant bacteria has further increased the difficulty of treatment (Kavanagh et al., 2018). Osteomyelitis not only causes local pain, sinus tract formation, bone necrosis, and bone defects, but may also lead to prolonged hospitalization, repeated surgical procedures, and even amputation, imposing a substantial burden on patients. Epidemiological studies have shown that the disease burden of osteomyelitis has increased in recent years because of the growing prevalence of risk factors such as diabetes, vascular disease, and population aging, resulting in a considerable healthcare burden for patients (Lew and Waldvogel, 2004). In recent years, antibacterial polyphenols and biomaterials designed using antibacterial polyphenols have increasingly been applied in the treatment of osteomyelitis. This mini-review aims to summarize the antibacterial mechanisms of polyphenols and describe representative strategies for the treatment of osteomyelitis using antibacterial polyphenols (Figure 1).

## 2 The infectious microenvironment and antibacterial challenges in osteomyelitis

Current clinical treatment of osteomyelitis remains centered on surgical debridement combined with antibiotic therapy. Thorough removal of necrotic tissue, pus, and infected implants helps reduce the local bacterial burden, while systemic or local antibiotics are used to eliminate residual pathogens. In cases involving substantial bone defects, dead-space management, bone grafting, or staged reconstruction may also be required (Panteli and Giannoudis, 2016; Lari et al., 2024). However, osteomyelitis lesions often have poorly defined boundaries. Excessive debridement may enlarge the defect, whereas insufficient debridement may leave infected tissue behind. Systemic antibiotics are also limited by their penetration into bone tissue, local blood supply, and drug toxicity, while prolonged use may increase the risks of antibacterial resistance and microbiota dysbiosis (Spellberg and Lipsky, 2012). The refractory and recurrent nature of osteomyelitis is largely attributable to its distinctive infectious microenvironment. Bacteria can adhere to the surfaces of implants, sequestra, and the bone matrix and subsequently form biofilms. Extracellular polymeric substances within biofilms can restrict drug diffusion and maintain the embedded bacteria in a low-metabolic state, thereby reducing their susceptibility to conventional antibiotics (Hall and Mah, 2017). Some bacteria can also transition into small-colony variants (SCVs) characterized by slow growth, reduced metabolism, and intracellular persistence. *Staphylococcus aureus* may also invade osteoblasts, osteocytes, or macrophages and further colonize the osteocyte lacuno-canalicular network. The narrow spaces within this microstructure are difficult for immune cells and most antibacterial agents to access effectively and may therefore serve as important reservoirs for persistent infection and recurrence (Masters et al., 2019).

In addition, sequestra lack an effective blood supply, and the local environment is often characterized by hypoxia, acidification, nutrient deprivation, and persistent inflammation. High levels of inflammatory mediators and reactive oxygen species can damage osteoblasts and vascular endothelial cells and promote osteoclast activation, ultimately establishing a vicious cycle of “infection–inflammation–bone destruction–reduced blood supply” (Masters et al., 2022). Therefore, an ideal therapeutic strategy for osteomyelitis should not only eradicate planktonic bacteria, but also penetrate or disrupt biofilms, suppress metabolically inactive and intracellular bacteria, and simultaneously address inflammation control, angiogenesis, and bone tissue repair.

## 3 Classes, structural basis, and mechanisms of action of antibacterial polyphenols

Antibacterial polyphenols are natural polyphenols with direct bacteriostatic, bactericidal, or antibiofilm activities. Compared with conventional antibiotics, which generally act on a relatively limited range of targets, antibacterial polyphenols can simultaneously affect bacterial membranes, metabolic enzymes, and metal homeostasis. Common antibacterial polyphenols mainly include flavonoids, phenolic acids, stilbenes, and tannins. Quercetin and catechins are representative flavonoids, and their catechol and galloyl groups enhance redox activity, metal coordination, and interfacial adsorption. Phenolic acids, such as gallic acid, have relatively small molecular structures and favorable diffusibility and can readily participate in coordination or chemical grafting. Other polyphenols, such as tannic acid, have high molecular weights and abundant phenolic hydroxyl groups, providing both protein-binding and material-assembly capabilities. They are therefore commonly used to construct coatings and metal–polyphenol networks (Panche et al., 2016; Ejima et al., 2013). The antibacterial activity of polyphenols is closely related to their structures. The number and position of phenolic hydroxyl groups influence their hydrogen-donating capacity and determine their binding strength with membrane lipids, proteins, and metal ions. Aromatic rings and conjugated planar structures can increase membrane affinity, whereas excessive hydrophobicity may reduce dispersion in aqueous environments. Small-molecule polyphenols can more readily diffuse to the bacterial surface or into bacterial cells, whereas highly polymerized tannins, despite their stronger protein-binding capacity, may be limited in their ability to penetrate biofilms. Glycosylation or methylation can also alter solubility and membrane permeability; therefore, polyphenols within the same class may exhibit different levels of activity (Cushnie and Lamb, 2005). The cell membrane is one of the most common targets of antibacterial polyphenols. Polyphenols can insert into the lipid bilayer and interact with membrane proteins or cell-wall components, leading to membrane depolarization, increased permeability, and leakage of intracellular contents (Kajiya et al., 2004; Borges et al., 2013). After entering bacterial cells, some polyphenols can also bind to metabolic enzymes or nucleic acid-associated proteins, thereby interfering with energy metabolism, DNA replication, and cell division. Their chelation of iron, copper, or zinc ions can also disrupt bacterial metal homeostasis. Under specific conditions, polyphenol–metal complexes may further promote the generation of reactive oxygen species and enhance bactericidal activity (Li et al., 2022).

In addition to inhibiting planktonic bacteria, antibacterial polyphenols can affect bacterial adhesion and biofilm maturation. They can alter bacterial surface properties, weaken the binding of adhesins to the host matrix, and disrupt extracellular polymeric substances. Some polyphenols can also increase membrane permeability or inhibit efflux pumps, thereby increasing bacterial susceptibility to antibiotics (Trentin et al., 2015; Stenvang et al., 2016). These multitarget effects may be compatible with the coexistence of planktonic bacteria, biofilm-associated bacteria, and metabolically inactive persister cells in osteomyelitis. However, free polyphenols still have limitations, including poor solubility, low stability, and limited local retention. Material-based carriers are therefore required to improve their delivery and extend their anti-inflammatory and bone-repair functions.

### 3.1 Biomaterial-based construction and multifunctional enhancement of antibacterial polyphenols

Antibacterial polyphenols possess multitarget antibacterial and antibiofilm activities, but free polyphenol molecules are often limited by poor water solubility, low stability, and rapid metabolism *in vivo*. Osteomyelitis lesions are also characterized by impaired blood supply and restricted tissue diffusion, making it difficult to maintain effective local concentrations over prolonged periods. Combining polyphenols with biomaterials can not only improve local delivery but also exploit their molecular structures in material construction, thereby creating therapeutic systems with both antibacterial and tissue-regulatory functions.

### 3.2 Integration of antibacterial polyphenols with biomaterials

Polyphenol molecules contain abundant phenolic hydroxyl groups and aromatic structures, allowing them to interact with polymers or proteins through hydrogen bonding, hydrophobic interactions, and π–π interactions. Molecules containing catechol or pyrogallol groups also exhibit strong metal-coordination abilities and can rapidly assemble with iron, copper, or zinc ions to form metal–polyphenol networks. These systems can be prepared under mild conditions and used for particle coating, implant surface modification, and drug co-loading. Coordination bonds are dynamic and can dissociate in response to changes in acidic or redox conditions, making them suitable for constructing infection-responsive delivery systems (Ejima et al., 2013; Li et al., 2022). Polyphenols can also be combined with natural or synthetic polymers. Chitosan, gelatin, and silk fibroin have favorable biocompatibility and can load polyphenols through noncovalent interactions, whereas synthetic materials such as poly (lactic-co-glycolic acid) facilitate the regulation of degradation and release. These combinations can be formulated into nanoparticles, microspheres, or hydrogels. Physical encapsulation helps preserve polyphenol activity but may result in burst release. Covalent grafting can improve stability but may consume some phenolic hydroxyl groups and affect their original functions. Therefore, material design must balance loading stability with the preservation of biological activity.

Inorganic materials can likewise serve as carriers for polyphenols. Hydroxyapatite, calcium phosphate materials, and bioactive glass possess osteoconductive properties, and their surface sites can adsorb or coordinate with polyphenols, thereby supporting both local antibacterial activity and bone repair (Wu et al., 2022; Ranjbar et al., 2023). On implant surfaces, polyphenols can also form coatings through coordination-assisted deposition or layer-by-layer assembly. Antibiotics or functional ions can subsequently be immobilized within these coatings to reduce early bacterial adhesion and biofilm formation (Gomez-Florit et al., 2016; Llopis-Grimalt et al., 2020).

### 3.3 Multifunctional properties of biomaterial-integrated antibacterial polyphenols

Biomaterial integration first improves the stability and local retention of polyphenols. Nanoparticles can enhance the dispersibility of poorly soluble polyphenols, microspheres and hydrogels are suitable for filling irregular dead spaces after debridement, and implant coatings can concentrate therapeutic agents at bacterial adhesion interfaces. Sustained release can prolong effective exposure and prevent insufficient activity caused by the rapid diffusion of free polyphenols. Second, biomaterial systems can enhance antibacterial and antibiofilm effects. Polyphenols can disrupt bacterial membranes and extracellular polymeric substances, while carriers increase their accumulation at infected interfaces. Co-loading with antibiotics can enhance drug penetration into biofilms. Metal–polyphenol systems can also release antibacterial ions and promote reactive oxygen species generation under specific conditions. Some materials incorporate photothermal or photodynamic therapy to further disrupt mature biofilms (Ye et al., 2024; Wu et al., 2024). However, excessive levels of metal ions or reactive oxygen species may also damage host cells; therefore, the intensity of antibacterial activity must be carefully controlled. In addition to their antibacterial effects, polyphenol-based materials can regulate inflammation, oxidative stress, and bone metabolism. Polyphenols can modulate signaling pathways such as NF-κB and MAPK, thereby reducing damage to osteoblasts and vascular endothelial cells caused by persistent inflammation (Xu et al., 2021). When combined with calcium phosphate materials, bioactive glass, or three-dimensional scaffolds, their biological regulatory effects can act synergistically with the osteoconductive properties of the carriers, providing favorable conditions for cell migration and new tissue ingrowth. Some systems may also support angiogenesis by improving the local redox environment or introducing functional ions, although further *in vivo* evidence is required.

Overall, an ideal polyphenol-based biomaterial should possess temporally regulated functions. During the early stage of infection, the bacterial burden must be rapidly reduced through pro-oxidative strategies, photothermal effects, or synergy with antibiotics. Once the infection has been controlled, the functions of the material should gradually shift toward anti-inflammatory, antioxidant, and bone-regenerative activities. Such a stage-specific transition is more consistent with the therapeutic progression of osteomyelitis from infection control to defect repair. Therefore, biomaterial integration is not simply intended to increase polyphenol loading but rather to exploit their structural reactivity to integrate local delivery, microenvironmental responsiveness, and tissue repair within a single system.

## 4 Applications of antibacterial polyphenol–biomaterial systems in osteomyelitis treatment

**FIGURE 2:**
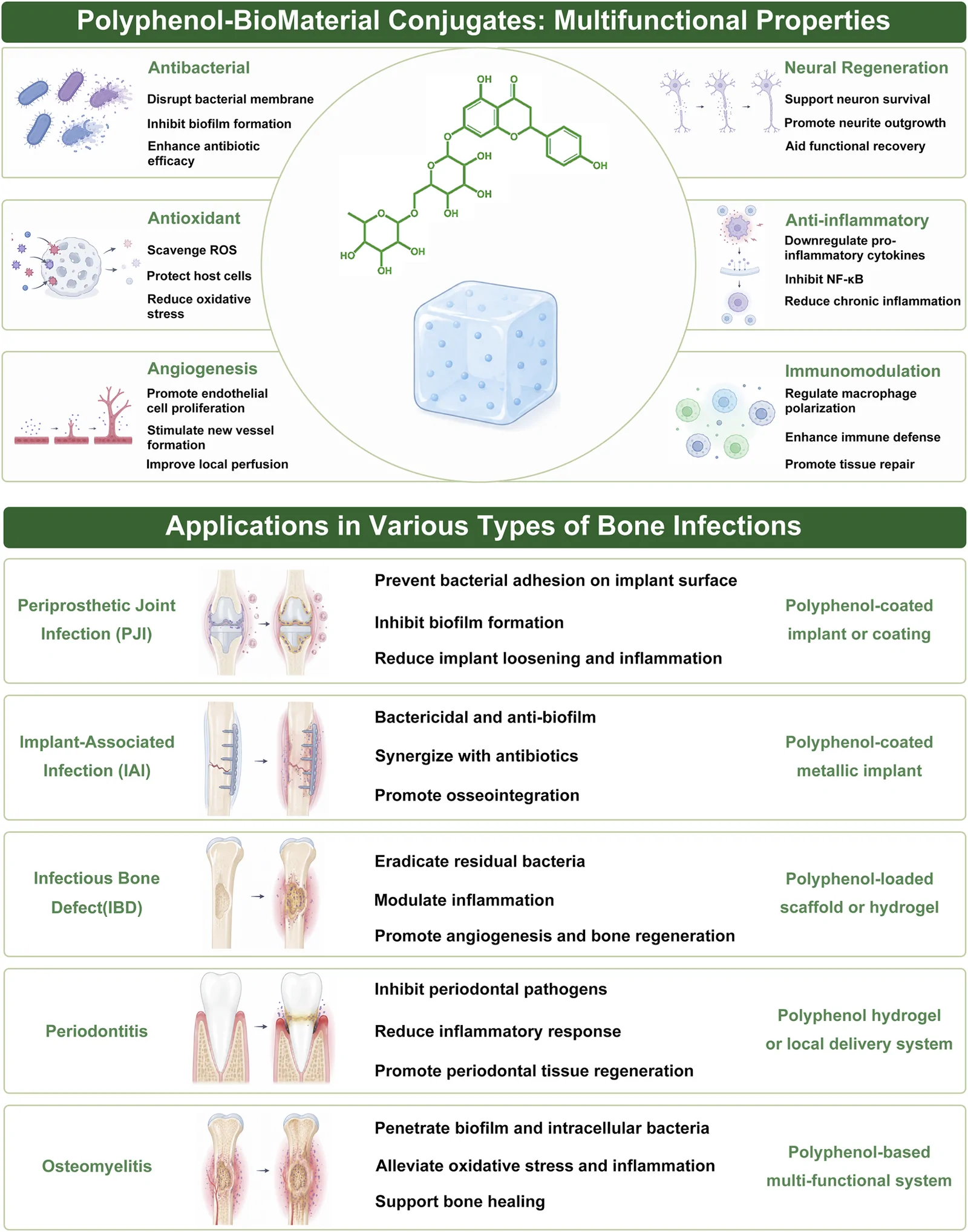
Multifunctional properties of polyphenol–biomaterial conjugates and their potential applications in bone-related infections.

The biomaterial-based construction strategies described above provide antibacterial polyphenols with tunable material forms, release profiles, and biological functions, thereby establishing a basis for their adaptation to the complex lesions of osteomyelitis (Figure 2). Osteomyelitis exhibits marked spatial heterogeneity and stage-dependent changes. Implant-associated infections are dominated by surface adhesion and biofilm formation; chronic infections are often accompanied by sequestra, deep bacterial reservoirs, and impaired local blood supply; and infected bone defects following debridement additionally require vascular and bone tissue reconstruction. Therefore, different carrier forms can be selected for antibacterial polyphenol–biomaterial systems according to lesion characteristics, while modulation of release rates and therapeutic timing may enable customized and adaptive treatment progressing from infection control to tissue repair (Geng et al., 2025).

### 4.1 Implant-associated infections and biofilm control

Bacterial adhesion to implant surfaces is the initial step in biofilm formation. Llopis-Grimalt and colleagues immobilized quercitrin on the surface of three-dimensional printed porous Ti-6Al-4V implants using a wet-chemical method to construct a quercitrin-functionalized coating. The coating reduced the adhesion and survival of *Staphylococcus epidermidis*, while promoting MC3T3-E1 cell adhesion and increasing alkaline phosphatase activity under lipopolysaccharide-stimulated conditions. These findings indicate that polyphenol coatings can inhibit early bacterial colonization while maintaining osteogenic activity (Llopis-Grimalt et al., 2020). Other studies have further employed layer-by-layer assembly to construct a (TA@HA/Lys) (n) composite coating composed of tannic acid, nano-hydroxyapatite, and lysozyme on titanium surfaces. In this system, tannic acid provides both interfacial adhesion and protein-binding functions, lysozyme exerts bactericidal activity by hydrolyzing bacterial cell walls, and hydroxyapatite provides an osteoconductive environment. The coating disrupted the membrane structures of *Staphylococcus aureus* and *Escherichia coli* and also exhibited antioxidant and osteogenic effects (Wang et al., 2021).

### 4.2 Deep-seated bacteria and persistent infection

Chronic osteomyelitis lesions are often accompanied by sequestra and mature biofilms, while impaired local blood supply limits the delivery of systemic drugs. To address this issue, quercetin was encapsulated in PLGA nanoparticles and co-integrated with vancomycin into a silk fibroin/chitosan–thiourea hydrogel scaffold for the treatment of chronic MRSA osteomyelitis in rats. In this system, PLGA nanoparticles delayed quercetin release, while vancomycin retained its direct bactericidal activity against MRSA, thereby establishing a localized combination-delivery system (Jafarbeglou et al., 2024). In another study, Cu^2+^ was coordinated with tannic acid to form copper–tannic acid nanosheets, which were further loaded with vancomycin to construct Van@CuTA nanocomposites. The results showed that the CuTA nanosheets delayed vancomycin release. Following treatment, local signs of infection, bone structural damage, and inflammatory infiltration of the bone marrow were all alleviated (Zhang et al., 2025). These studies provide direct animal evidence supporting the use of polyphenol coordination materials for chronic osteomyelitis treatment.

### 4.3 Regulation of the infectious microenvironment and combination therapy

Acidification and redox imbalance within osteomyelitis lesions can serve as triggers for material responsiveness. One related system utilized the dynamic dissociation of metal–polyphenol coordination bonds under acidic conditions to achieve sustained drug release. Its release behavior at pH 5.0 was well matched to the acidic local characteristics of chronic bone infection (Zhang et al., 2025). Regarding energy-responsive therapy, Ye and colleagues prepared EGCG–Au nanoparticles through the one-step self-assembly of EGCG and gold ions. Following near-infrared irradiation, the system synergistically disrupted MRSA and its biofilms through mild photothermal effects, reactive oxygen species generation, and quinoprotein formation, while also affecting bacterial virulence-related genes (Ye et al., 2024). This study demonstrates that polyphenol–metal systems can integrate antibacterial, pro-oxidative, and inflammation-regulatory functions within a single platform.

### 4.4 Integrated repair of infected bone defects

Materials for infected bone defects must not only reduce the bacterial burden but also provide space and biological activity for new bone formation. In one study, gallic acid, pyrogallol, and tannic acid were separately used as liquid-phase components and mixed with bioactive calcium silicate bone cement to construct polyphenol-modified bone cements. The incorporation of polyphenols significantly enhanced the inhibitory effects of the materials against *S. aureus* and *E. coli* (Wu et al., 2022). In another study, 58S bioactive glass was used as the scaffold substrate and coated with kaempferol-loaded zein. The results showed that kaempferol increased cellular alkaline phosphatase activity, calcium deposition, and the expression of osteogenesis-related genes. In a rat critical-sized calvarial defect model, scaffolds loaded with kaempferol and stem cells achieved the most pronounced new bone formation at 12 weeks (Ranjbar et al., 2023).

In addition to providing sustained vancomycin release and inhibiting MRSA, some materials promoted the mineralization of bone marrow mesenchymal stem cells as well as the migration and tube formation of vascular endothelial cells, and significantly reduced bone destruction in a chronic osteomyelitis model (Zhang et al., 2025). These findings suggest that antibacterial polyphenol–biomaterial systems may coordinate local antibacterial activity, angiogenesis, and bone regeneration. However, their actual therapeutic efficacy in large infected segmental bone defects remains insufficiently validated and represents a potential direction for future research.

## 5 Conclusion

Natural polyphenols offer a promising platform for osteomyelitis treatment because their antibacterial and antibiofilm activities can be combined with local delivery and bone repair. Their phenolic hydroxyl groups and metal-coordination ability enable incorporation into nanoparticles, hydrogels, implant coatings, and bone scaffolds, supporting local retention, controlled release, and combination therapy. However, major barriers remain before clinical translation. The structure–activity relationships, primary antibacterial targets, effective *in vivo* doses, pharmacokinetics, and tissue distribution of many polyphenols are still unclear. Direct evidence against mature biofilms, SCVs, intracellular bacteria, persister cells, and bacteria within the osteocyte lacuno-canalicular network is limited. Differences in bacterial strains, animal models, treatment protocols, and outcome measures also hinder cross-study comparison. In addition, the long-term safety of metal-ion release, reactive oxygen species generation, degradation products, and prolonged implantation requires systematic evaluation. Batch variability of natural polyphenols, sterilization and storage stability, scalable manufacturing, quality control, and regulatory approval further complicate clinical development. Future studies should establish quantitative structure–activity and dose–response relationships, develop standardized osteomyelitis models, and assess long-term recurrence, bone healing quality, systemic safety, and material degradation. Direct comparison with standard debridement and antibiotic therapy is also necessary to determine the additional clinical value of these systems. With improved reproducibility and disease-specific validation, polyphenol-based biomaterials may help integrate infection control with bone regeneration.
